# A Prospective Trial With Ketoconazole Induction Therapy and Octreotide Maintenance Treatment for Cushing's Disease

**DOI:** 10.1210/jendso/bvaf089

**Published:** 2025-05-23

**Authors:** Ticiana Paes, Rob van der Pas, Peter M van Koetsveld, Fadime Dogan, Kees K A van den Berge, Romana T Netea-Maier, Peter H Bisschop, Leo J Hofland, Richard A Feelders

**Affiliations:** Department of Internal Medicine, Division of Endocrinology, Erasmus MC Cancer Institute, Erasmus Medical Center, Rotterdam 3015 GD, The Netherlands; Department of Internal Medicine, Roger Williams Medical Center, Providence, RI 02908, USA; Department of Internal Medicine, Bernhoven Hospital, Uden 5406 PT, The Netherlands; Department of Internal Medicine, Division of Endocrinology, Erasmus MC Cancer Institute, Erasmus Medical Center, Rotterdam 3015 GD, The Netherlands; Department of Internal Medicine, Division of Endocrinology, Erasmus MC Cancer Institute, Erasmus Medical Center, Rotterdam 3015 GD, The Netherlands; Department of Internal Medicine, Admiraal de Ruyter Hospital, Goes 4462 RA, The Netherlands; Department of Internal Medicine, Division of Endocrinology, Radboud University Medical Center, Nijmegen 6525 GA, The Netherlands; Department of Endocrinology and Metabolism, Amsterdam University Medical Center, Amsterdam 1105 AZ, The Netherlands; Department of Internal Medicine, Division of Endocrinology, Erasmus MC Cancer Institute, Erasmus Medical Center, Rotterdam 3015 GD, The Netherlands; Department of Internal Medicine, Division of Endocrinology, Erasmus MC Cancer Institute, Erasmus Medical Center, Rotterdam 3015 GD, The Netherlands; Holman Division of Endocrinology, Diabetes and Metabolism, New York University Langone Medical Center, New York, NY 10016, USA

**Keywords:** Cushing’s disease, ketoconazole, octreotide, somatostatin receptor, cortisol

## Abstract

**Context and Objective:**

The lack of efficacy of somatostatin receptor subtype 2 (SST2) preferring somatostatin analogs in patients with Cushing's disease (CD) results from a downregulating effect of hypercortisolism on SST2 expression. Our objective is to evaluate the efficacy of a strategy with sequential treatment of ketoconazole to reduce cortisol levels and potentially restore SST2 expression followed by octreotide as maintenance therapy in patients with CD.

**Patients and Design:**

Fourteen adult patients with CD were prospectively enrolled. Patients started with ketoconazole. Once cortisol levels were normalized, octreotide was initiated. After 2 months of combination therapy, patients were maintained on octreotide monotherapy until the end of the study period (9 months). Treatment success was defined by normalization of urinary free cortisol (UFC) levels.

**Results:**

Ketoconazole was able to normalize UFC levels in 11 (79%) patients. Octreotide effectively sustained normal levels of UFC in 3 patients (27%) (responders). Four patients (36%) showed a partial response. The remaining 4 (36%) patients developed hypercortisolism as soon as ketoconazole was stopped (nonresponders). Octreotide responders had lower UFC levels at baseline when compared to partial responders and nonresponders (1.40 ± 0.07 vs 2.05 ± 0.20 UNL, *P* = 0.083). SST2 mRNA was highly expressed in adenomas of 2 responder patients (0.803 and 0.216 copies per *hprt*).

**Conclusion:**

Sequential treatment with ketoconazole to lower cortisol levels followed by octreotide to maintain biochemical remission according to UFC may be effective in a subset of patients with mild CD, suggesting that cortisol-mediated suppression of SST2 expression is a reversible process.

Transsphenoidal adenomectomy is the first-line treatment of Cushing's disease (CD) [1-3]. Medical therapy can be used as an adjunctive preoperative treatment or in persistent or recurrent disease [2, 4, 5]. Pharmacological treatment of CD can be divided into 3 approaches: pituitary-directed therapy, steroids synthesis inhibitors, and glucocorticoid receptor antagonists [4]. Because of limited efficacy and side effects, a combination of drugs is often necessary to achieve biochemical control [2, 5-8].

Steroid synthesis inhibitors are often used as a first-line medical treatment modality. Ketoconazole and metyrapone can normalize cortisol production in about 50% to 60% of patients, whereas the recently introduced steroidogenic enzyme inhibitor osilodrostat has an efficacy of up to 80% [9-11]. Pharmacotherapy targeting the corticotroph tumor itself may be a more rational approach since it exerts effects at the cause of the disease [2, 5, 12]. The most commonly used drugs in this category are cabergoline, a dopamine agonist, and pasireotide, a second-generation somatostatin analog [2, 3, 13]. Cabergoline inhibits ACTH secretion through agonism of the dopamine type 2 receptor, expressed in the majority of corticotroph tumors [14, 15]. However, cabergoline is able to normalize the cortisol secretion in less than half the patients, and a substantial number of patients escape from treatment [4, 8, 16, 17]. Several small studies show promising effects of cabergoline combined with ketoconazole [7, 8]. Pasireotide exhibits high-affinity binding to somatostatin receptor subtype (SST) 5, which is the SST expressed at the highest level in corticotroph pituitary adenomas. Pasireotide shows moderate efficacy in normalizing cortisol levels in a subset of patients with mild to moderate hypercortisolism, with hyperglycemia as an important side effect [13, 18, 19].

Octreotide, a somatostatin analog with high binding affinity to SST2, was shown to lower ACTH production in patients with corticotroph tumor progression following bilateral adrenalectomy but was unsuccessful in patients with active CD [20, 21]. Table 1 provides an overview of the clinical studies using octreotide in CD. Tumoral pituitary corticotroph cells express about 5 to 10 times higher SST5 compared to SST2, which may explain the reduced efficacy of octreotide compared to pasireotide in inhibiting ACTH secretion in primary cultures of human corticotroph tumors as well as in vivo [13, 28]. This is explained by selective suppressive effects of high cortisol concentrations in active CD on SST2 expression, resulting in an absent treatment response to octreotide [13, 29, 30]. Hence, it may be hypothesized that normalizing or lowering cortisol levels in patients with CD can result in a reciprocal increase in SST2 expression by corticotroph tumor cells. Under such conditions, the use of octreotide could play a potential role in CD management based on its safer toxicity profile compared to pasireotide [31].

**Table 1.** Literature review: octreotide treatment in patients with Cushing's disease

Study	n	Maximal octreotide dose	Response criteria	Full response	Partial response	No response	Maximal treatment duration
Invitti et al, 1990 [22]	3	1200 µg/day	UFC		1	2	49 days
Lamberts et al, 1989 [20]	3	100 µg (single injection)	Serum cortisol			3	Trial 12 hours
Arregger et al, 2012 [21]	2	Oct-lar (20 mg/month)	UFC			2	4 months
Woodhouse et al, 1993 [23]	4	100-500 µg (every 8 hours)	Serum cortisol			4	Trial 24-72 hours
El-Shafie et al, 2015 [24]	6	100 µg (every 8 hours)	Serum cortisol			6	Trial 72 hours
Ambrosi et al, 1990 [25]	4	100 µg (single injection)	Serum cortisol			4	Trial 7 hours (CRH stimulus)
Stalla et al, 1994 [26]	5	100 µg (30 and 180 minutes)	serum cortisol			5	Trial 400 minutes (CRH stimulus)
Vignati et al, 1996 [27]	3	100 µg (single injection)/300 µg/day	Serum cortisol/UFC		1	2	Trial 8 hours/75 days
Total	30			0	2 (7%)	28	

Abbreviation: Oct-lar, long acting repeatable octreotide; UFC, urinary free cortisol.

We previously demonstrated that in corticotroph adenomas obtained from CD patients who were in biochemical remission before surgery, induced by medical treatment, *SST2* mRNA expression was significantly higher compared to corticotroph tumor tissue from patients with hypercortisolism at the time of operation [32]. In fact, *SST2* mRNA levels in adenomas from these normocortisolemic patients were comparable to those of GH-producing adenomas, which are usually responsive to SST2-preferring somatostatin analogs [32]. In this pilot study, we, therefore, aim to evaluate the clinical efficacy of a sequential regimen with ketoconazole induction therapy to reduce cortisol levels in CD and potentially restore SST2 expression at the level of the corticotroph adenoma, followed by octreotide treatment to reduce ACTH secretion.

## Methods

### Study Population

Adult patients with recently diagnosed treatment-naïve CD or with persistent or recurrent hypercortisolism after transsphenoidal surgery were eligible for enrollment. Patients already on medical treatment for CD were included only after a drug washout period of 4 weeks and following confirmation of hypercortisolism. Exclusion criteria included elevated liver enzymes, renal insufficiency, history of pituitary radiotherapy, symptomatic cholelithiasis, and pregnancy.

The study protocol was approved by the ethical committees of the participating centers. All patients gave their written informed consent. The trial was registered by the Dutch Trial Register (nr. NL37105.078.11).

### Diagnostic Workup of CD

Upon clinical evidence of CD, the diagnosis was biochemically established by elevated 24-h urinary free cortisol (UFC) concentrations (3 samples), failure in suppressing plasma cortisol after 1 mg of dexamethasone, and increased midnight saliva cortisol levels. ACTH dependency was defined on the basis of normal to high ACTH plasma levels. Additionally, plasma cortisol diurnal rhythm was assessed with measurement at 9 Am, 5 Pm, 10 Pm, and midnight. Once a diagnosis of ACTH-dependent hypercortisolism was confirmed, magnetic resonance imaging was performed to detect a pituitary tumor. In the absence of a lesion, or a lesion of less than 6 mm, bilateral inferior petrosal sinus sampling was performed to confirm central ACTH overproduction.

### Drug Regimen Protocol

**Figure 1.**
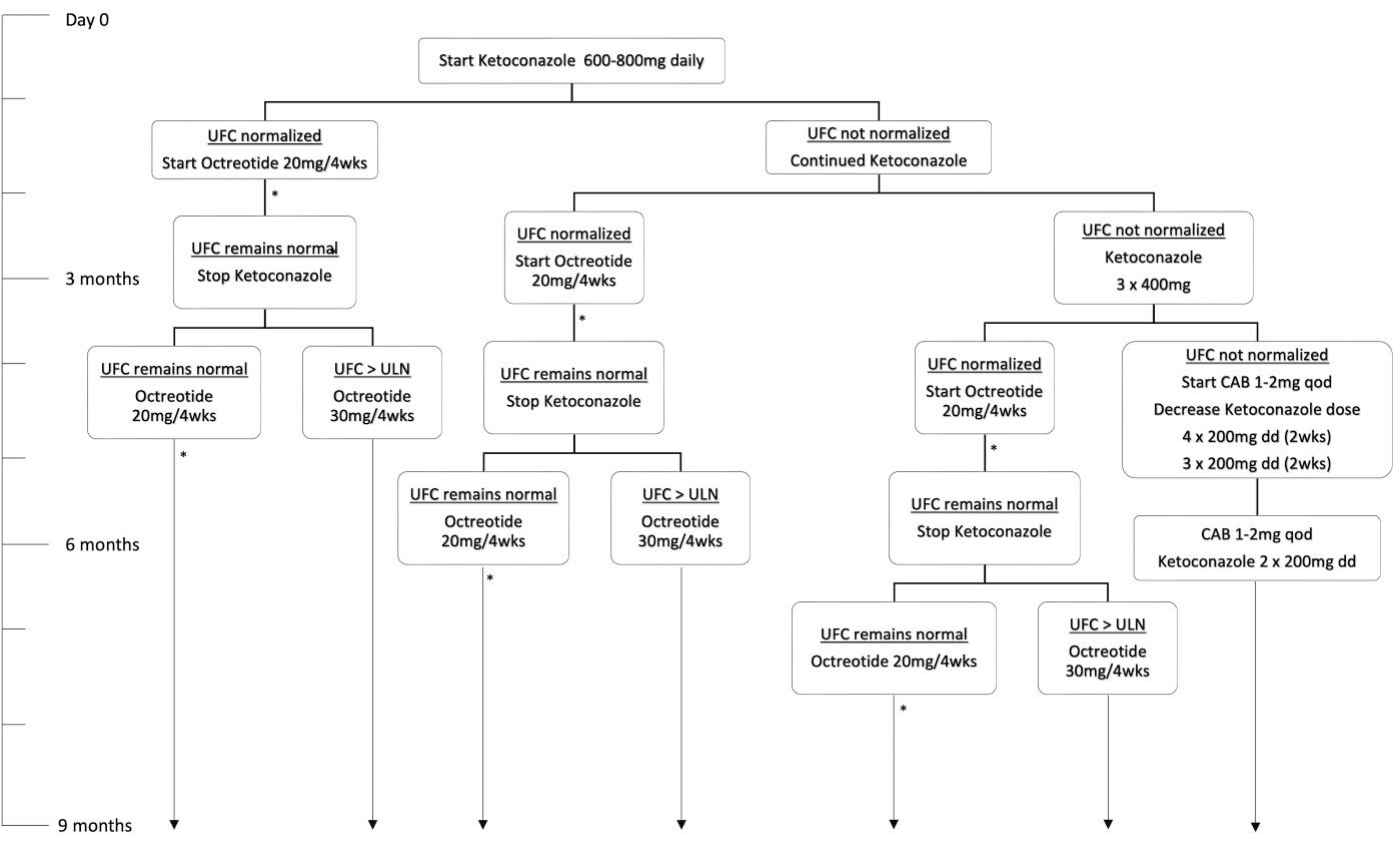
Study protocol. If UFC excretion (mean of 2 collections) increases again (above the ULN) under octreotide/ketoconazole combination therapy or octreotide monotherapy (20 mg every 4 weeks), the octreotide dosage will be increased to 30 mg every 4 weeks. Abbreviations: CAB, cabergoline; UFC, urinary free cortisol.; ULN, upper limit of normal.

After inclusion, patients were followed monthly for up to 9 months. All patients started with ketoconazole; the initial dose depended on the severity of hypercortisolism, with 600 mg per day for mild hypercortisolism [UFC ≤ 1.5 times the upper limit of normal (ULN)] and 800 mg per day for a higher level of hypercortisolism (UFC >1.5 times the ULN). (Fig. 1). If necessary, the dose of ketoconazole could be uptitrated to 1200 mg per day after 2 months to achieve biochemical remission according to UFC excretion. Once UFC levels were normalized, long acting repeatable (LAR) octreotide treatment was initiated at a dose of 20 mg every 4 weeks. If UFC concentrations remained normal after 2 months of combined therapy (ketoconazole plus octreotide), ketoconazole was discontinued and patients were maintained on octreotide monotherapy until the end of the study period. If the UFC level (mean of 2 samples) was increased above the ULN, the octreotide dose was increased from 20 to 30 mg every 4 weeks. This may have occurred earlier, while octreotide was still combined with ketoconazole, or later, on octreotide monotherapy.

Response to octreotide was defined as the maintenance of normal UFC levels after ketoconazole discontinuation until the end of the study period, while partial response was defined as normal UFC levels maintained for at least 1 month after ketoconazole discontinuation and/or a >50% decrease of UFC levels at the last follow-up visit compared to the baseline value. Lack of response to octreotide was defined by the inability of octreotide to maintain normal UFC levels after discontinuation of ketoconazole. In this respect, a persistently elevated UFC concentration for 2 consecutive months was considered as treatment failure, after which the study protocol was terminated earlier, before the study period of 9 months. In case of biochemical remission, octreotide monotherapy was maintained until the end of the study period of 9 months, after which octreotide could be continued or replaced by another treatment modality.

In case ketoconazole therapy for 3 months failed to control cortisol production, a different treatment regimen was introduced. Cabergoline instead of octreotide was added to ketoconazole in an attempt to achieve biochemical control. Cabergoline, starting at 0.5 mg every other day, was gradually increased up to 1 and eventually 2 mg every other day, as needed, and ketoconazole was gradually reduced from 1200 to 400 mg per day within 4 weeks. If successful, this combination treatment (ketoconazole and cabergoline) was maintained until the end of the study period.

### Side-effects Monitoring

Between the visits, patients were contacted by telephone for monitoring of adverse events. At each visit, laboratory evaluation was performed of pituitary function, hematology, blood chemistry, liver enzymes and renal function, hemoglobin A1c, glucose, and insulin levels.

During treatment with ketoconazole, concentrations of liver enzymes (aspartate transaminase, alanine transaminase, alkaline phosphatase, and gamma glutamyl transferase) were regularly measured. In case of an increase in liver enzymes (>4x ULN), the ketoconazole dose was decreased by 50%. If dose reduction did not lead to normalization of liver enzyme concentrations, ketoconazole was stopped with termination of the study. If relative adrenal insufficiency developed with steroid withdrawal complaints, the cortisol-lowering medication was stopped and eventually restarted at a lower dose. In case of absolute adrenal insufficiency hydrocortisone replacement therapy was started in addition to interruption of study medication. Electrocardiography was performed at baseline and at follow-up visits.

### Assessment of Treatment Efficacy

Twenty-four-hour urinary cortisol excretion (2 collections) was measured at each monthly visit. Plasma cortisol diurnal rhythm (CDR) was assessed at baseline and at 3, 6, and 9 months. Recovery of CDR was defined by a serum cortisol concentration at midnight of less than 67% of that at 0900 hours (Pm/am ratio >0.67) [33]. Biochemical remission was defined as normalization of UFC concentrations, ie, the mean of 2 collections below the ULN.

### Assessment of Clinical Parameters

Physical examination including measurement of blood pressure, heart rate, weight, height, body mass index, and waist circumference was performed at baseline and assessed monthly. Additionally, a routine laboratory examination, including full blood count, electrolytes, creatinine, blood urea nitrogen, liver enzymes, lipase, amylase, bilirubin, glucose, insulin, and glycosylated hemoglobin, was conducted at each visit.

### Quantitative PCR

Eleven patients underwent surgery after the study period. In 4 patients, sufficient corticotroph pituitary adenoma tissue was available to assess *SST2* mRNA expression. To assess the purity of the samples, *GH* mRNA relative to pro-opiomelanocortin (*POMC*) mRNA was calculated. Only samples with a GH/POMC ratio less than 10% for normal pituitary tissue were used in this analysis [34].

Quantitative PCR was performed following a protocol as previously described [35]. Briefly, poly A^+^ mRNA was isolated from corticotroph adenoma cells using oligo (dT)_25_ dynabeads (Invitrogen, Breda, The Netherlands). Subsequently, 23 µL H_2_O was added for elution, and 10 µL of poly A mRNA was used to synthesize cDNA using a commercial RevertAid First Strand cDNA synthesis kit (Thermo Scientific, Breda, The Netherlands). The assay for RT-qPCR was performed using Taqman Universal PCR mastermix (Applied Biosystems, Breda, The Netherlands) supplemented with sst2 forward and reverse primers and probes. (Supplementary Table S1) [36]. The expression of *SST2* mRNA was determined relative to the hypoxanthine phosphoribosyltransferase (*HPRT*) housekeeping gene.

### Immunohistochemistry

From 4 patients, representative adenoma tissue was available for immunohistochemistry (IHC). IHC was performed on 4-µm thick whole slide sections from formalin-fixed paraffin-embedded tissue blocks, on a validated and accredited automated slide stainer (Benchmark ULTRA System, VENTANA Medical Systems, Tucson, AZ, USA) according to the manufacturer's instructions. Briefly, following deparaffinization and heat-induced antigen retrieval, the tissue samples were incubated with rabbit anti-SST2A antibody (Biotrend; NB-49-015-1ML, dilution 1:25) for 32 minutes at 37°C, followed by Optiview detection (#760-500 and #760-700, Ventana). Counterstain was done by hematoxylin II for 12 minutes and a blue coloring reagent for 8 minutes. Each tissue slide contained a fragment of formalin-fixed paraffin-embedded pancreatic tissue as an on-slide positive control. A semiquantitative immunoreactivity scoring system (IRS) was used by 2 independent investigators to assess SST2 immunostaining. IRS is based on 2 scales: first, the fraction of positive-stained cells > 80%, 51% to 80%, 10% to 50%, <10% and 0 and second, the intensity of immunostaining as strong, moderate, weak, and negative. The product of these 2 factors was used to calculate the IRS final score (range from 0 to 12) [37].

### Statistical Analysis

Given the proof-of-concept nature of the present study, no formal statistical power and sample size calculations were performed. Patients were grouped according to the level of response to treatment in responders, partial responders, and nonresponders. For statistical comparisons, partial responders and nonresponders were grouped together and compared to responders.

Continuous variables are expressed as mean ± SEM. Categorical variables are expressed as counts and percentages. For comparisons between groups, Student's *t*-test was used. For paired comparisons (baseline vs follow-up), paired *t*-test was used. Statistical significance was set at *P* < .05. GraphPad Prism version 5.01 was used for statistical analysis.

## Results

### Study Population

Sixteen patients with CD were prospectively enrolled, of whom 14 started the study protocol. One patient withdrew at baseline, and 1 patient was excluded because of pseudo-Cushing's syndrome due to a psychiatric disorder. The mean age was 48.6 years; 64% (n = 9) were female; 86% (n = 12) were newly diagnosed and naïve in treatment; and 71% (n = 10) presented with mild hypercortisolism, defined as a UFC level <2 times the ULN, at baseline. The average treatment duration in this study was 6 months. Hypertension was the most common comorbidity (93%), followed by diabetes mellitus (50%) and dyslipidemia (43%). The majority of patients (79%, n = 11) exhibited a flattened cortisol rhythm with persistently high levels of plasma cortisol throughout the day (Table 2).

**Table 2.** Baseline demographic and clinical characteristics of the study population

Characteristics	Population (n = 14)
Female sex, no. (%)	9 (64.28)
Age at study, mean (median), years	48.64 (48)
Status of CD, no. (%)	
Newly diagnosed	12 (86)
Persistent	1 (7.1)
Recurrent	1 (7.1)
UFC level, times ULN, mean (median)	1.84 (1.76)
ACTH, mean, pg/mL	10.23 ± 6.8
Severity of CD, no. (%)*^a^*	
Mild	10 (71.42)
Moderate	4 (28.57)
Severe	0 (0)
Disturbed circadian diurnal rhythm, no. (%)*^b^*	11 (78.6)
Months of study completed, mean (median)	6.43 (7)
MRI, no. (%)	
Nonvisible adenomas	3 (21)
Microadenomas	9 (64)
Macroadenomas	2 (14)
Comorbidities, no. (%)	
Diabetes	7 (50)
Hypertension	13 (92.85)
Heart/vascular disease	3 (21.42)
Dyslipidemia	6 (42.85)
Obesity	5 (35.71)

Abbreviations: CD, Cushing’s disease; MRI, magnetic resonance imaging; UFC, urinary free cortisol; ULN, upper limit of normal.

^a^Mild hypercortisolism was defined as UFC level less than 2 times the ULN, moderate hypercortisolism as UFC level between 2 and 5 times the ULN, and severe hypercortisolism as UFC level above 5 times the ULN.

^b^Disturbed circadian diurnal rhythm was defined as serum cortisol concentration at 2400 hours/serum cortisol concentration at 0900 hours (Pm/am ratio) above 0.67 [33].

### Ketoconazole Treatment

All patients started treatment with ketoconazole monotherapy at a dose of 600 to 800 mg per day depending on baseline UFC level. In 11 patients (79%), normal values of UFC were achieved after 1 or 2 months of treatment. One patient developed symptoms of hypocortisolism with nausea, vomiting, and dizziness. Ketoconazole was discontinued and restarted a week later with a lower dose (200 mg/day), also resulting in normal UFC levels. Another patient discontinued the treatment in the first week because of clinical intolerance. A transient increase in liver enzymes was observed in 5 patients (39%), but no patient had to stop the study protocol because of liver toxicity. Most patients who achieved normal values of UFC (n = 11 out of 14; 79%) lost weight (mean weight loss = 7 ± 4.6 kg) during ketoconazole treatment. No abnormalities were found on electrocardiography during treatment with ketoconazole and octreotide mono- or combination therapy.

According to the study protocol, octreotide (20 mg every 28 days) was added to ketoconazole in the 11 patients who achieved normal cortisoluria. With the combination treatment, 9 patients (82%) sustained normal UFC levels. In 2 patients with recurrent hypercortisolism, increasing the dose of octreotide from 20 to 30 mg/4 weeks normalized UFC levels. Ketoconazole treatment was then stopped, and all patients continued octreotide (20 or 30 mg per month) monotherapy.

### Octreotide Treatment

**Figure 2.**
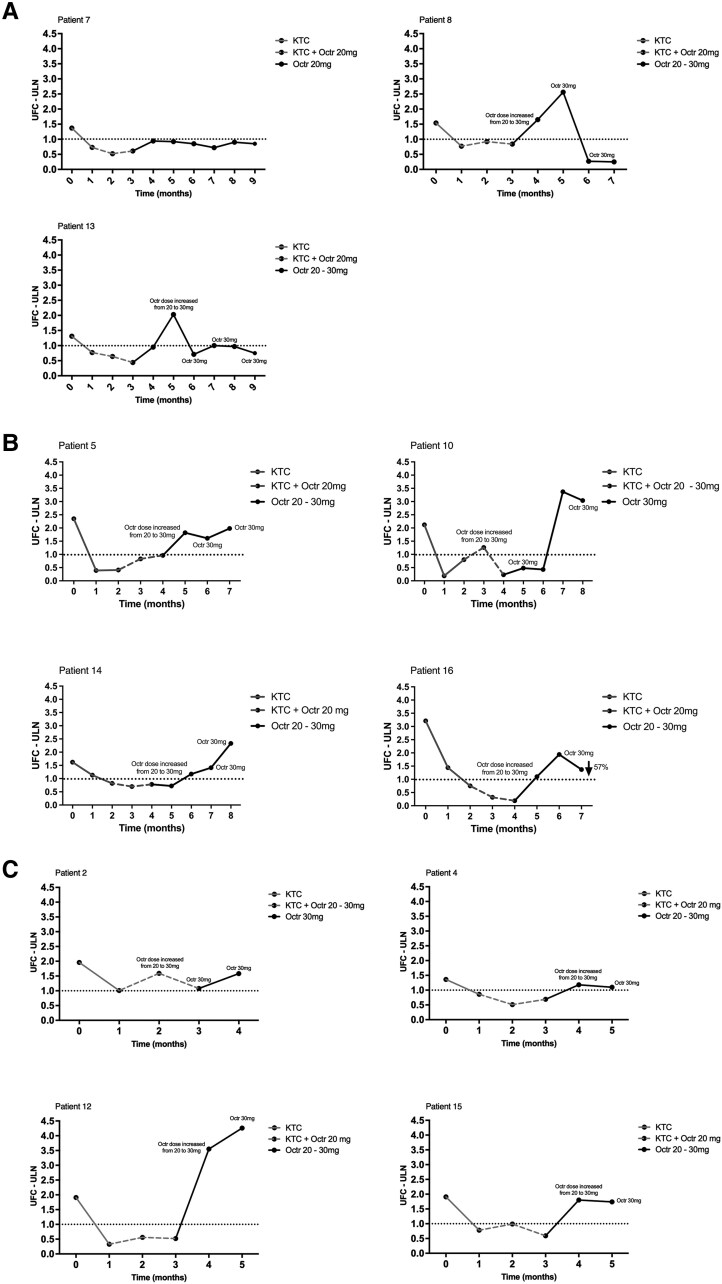
Levels of UFC under sequential KTC and Octr treatment. (A) Octr responders (n = 3, patients 7, 8, 13). All patients started treatment with KTC monotherapy at a dose of 600 mg per day. Subsequently, Octr (20 mg every 28 days) was added to the treatment regimen. After 2 months of combined therapy, KTC was discontinued. In 2 cases, this led to a gradual increase in UFC levels requiring a higher dose of Octr (30 mg/month). All 3 patients then remained in remission under Octr monotherapy. (B) Octr partial responders (n = 4, patients 5, 10, 14, and 16). The patients followed different treatment schedules. Patient 5 started with KTC monotherapy followed by 1 month of combined treatment (KTC + Octr) and subsequent Octr monotherapy. Under Octr treatment, the patient was in remission for 2 months. Patient 10 started with KTC monotherapy, followed by 3 months of combined treatment (KTC + Octr) because of an escape of the treatment requiring an increase in the dose of Octr from 20 to 30 mg/month and subsequently went on Octr 30 mg/month monotherapy. Under Octr treatment, the patient was in remission for 2 months. Patient 14 started with KTC monotherapy, achieving remission of the disease in the second month, followed by 2 months of combined treatment (KTC + Octr) and subsequent Octr monotherapy. Under Octr treatment, the patient was in remission for 1 month. The last patient (no. 16) started with KTC monotherapy, achieving a normal cortisol level, followed by combined treatment and subsequent Octr monotherapy. UFC levels at follow-up had decreased by 57% compared to baseline. (C) Octr nonresponders (n = 4, patients 2, 4, 12, and 15). All patients started treatment with KTC monotherapy at a dose of 600 to 800 mg per day. Subsequently, Octr was added to the treatment for 2 months. KTC was discontinued in the third month, which led to a gradual increase in UFC levels. Despite the increased dose of Octr (30 mg/month), all patients failed to maintain disease remission. Data represent mean ± SEM. Abbreviations: KTC, ketoconazole; Octr, octreotide; UFC, urinary free cortisol (24 hours).

Octreotide monotherapy maintained normal levels of UFC in 3 patients (27%) (responders, Fig 2A). Four (36%) other patients showed a partial response to octreotide (Fig. 2B shows the responses in the individual patients). In 3 of these patients, normal UFC levels were sustained for 1 or 2 months following discontinuation of ketoconazole, and in the other partial responder, the UFC levels at the last follow-up visit had decreased by 57% compared to the baseline levels. The remaining 4 patients developed hypercortisolism as soon as ketoconazole was stopped (nonresponders, Fig. 2C). Responders to octreotide monotherapy had lower UFC levels at baseline when compared to partial responders and nonresponders, with a trend to statistical significance (*P* = .083) (Table 3). No differences were observed between the 2 groups (responders vs partial responders and nonresponders) related to age, sex, number of comorbidities, and baseline and follow-up cortisol diurnal rhythm (Table 3).

**Table 3.** Clinical characteristics of responder compared to partial/nonresponder patients

Characteristics	Responders	Partial/nonresponders	*P*-value
No. of patients	3	8	
Age (years) (mean ± SEM)	39.67 ± 6.88	52 ± 4.30	.163
Number of comborbidities (mean ± SEM)	2.33 ± 0.33	2.38 ± 0.57	.967
Initial UFC (mean ± SEM)	1.40 ± 0.07	2.05 ± 0.20	.083
Baseline CDR, Pm/am ratio (mean ± SEM)	0.85 ± 0.14	0.91 ± 0.10	.752
Follow-up CDR, Pm/am ratio (mean ± SEM)	0.61 ± 0.17	0.81 ± 0.11	.43

Abbreviations: CDR, circadian diurnal rhythm; UFC, urinary free cortisol.

### Responders

**Figure 3.**
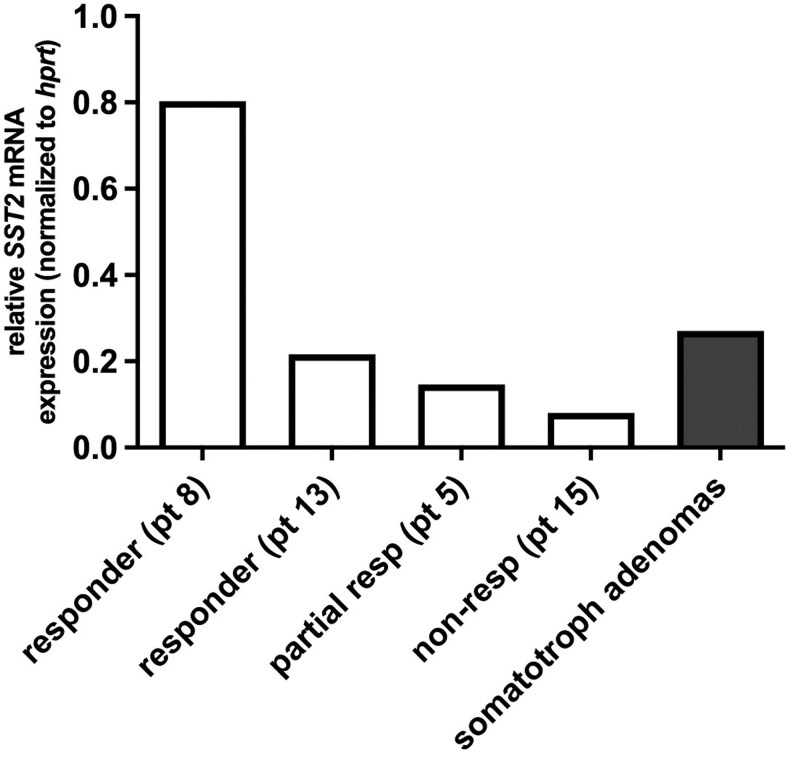
mRNA expression level of *SST2* in corticotroph tumors. *SST2* mRNA expression in responder (n = 2), partial responder (n = 1), and nonresponder (n = 1). *SST2* mRNA expression level in somatotroph tumors (filled bar) was included for comparison (n = 10; ratio over HPRT, mean ± SEM: 0.27 ± 0.08), as published previously by our group using a similar protocol [32]. Abbreviations: HPRT, hypoxanthine phosphoribosyltransferase; non-resp, nonresponder; partial resp, partial responder; pt, patient.

Individual patient numbers in brackets refer to the patient numbers in Figs. 2 and 3 and Supplementary Table S1 [36]. In 2 (patients 8 and 13) of the 3 responders, UFC levels gradually increased after discontinuation of ketoconazole treatment, requiring an increase in the octreotide dose from 20 to 30 mg that ultimately induced sustained normalization of UFC levels (Fig. 2A). Overall, among responders, the mean UFC levels at baseline was 1.40 ± 0.07 times the ULN and 0.62 ± 0.19 times the ULN at follow-up at the end of the study period (*P* = .09). Regarding the CDR, 2 patients (no. 7 and 13) at baseline exhibited disturbed CDR, and in 1 patient (no. 8), it was slightly altered. Full recovery of the CDR at follow-up was observed in 2 patients (no. 7 and 8), including the 1 (no. 8) with discrete alteration, while in another (patient 13), there was a partial recovery. On average, patients exhibited a numerically lower cortisol Pm/am ratio at follow-up as compared to baseline (baseline Pm/am ratios 0.86 ± 0.14 and 0.62 ± 0.09 at follow-up, *P* = .15). In terms of clinical features of CD, 2 (no. 7 and 13) of the 3 patients showed improvement in weight, waist circumference, and systolic and diastolic blood pressure during the treatment period, with the remaining patient (no. 8) showing a worsening of these parameters (Supplementary Table S2) [36].

### Partial Responders

Among the 4 patients (patients 5, 10, 14, and 16) with a partial response to octreotide monotherapy, UFC levels were sustained for 1 to 2 months in 3 patients with a gradual increase after ketoconazole discontinuation (Fig. 2B). In another patient, UFC levels at follow-up had decreased by at least 50% compared to baseline, albeit still at abnormal levels (Fig. 2B, patient 16). For all 4 patients, the mean UFC at baseline was 2.32 ± 0.33 and 2.18 ± 0.34 times the ULN at follow-up at the end of the study period (*P* = .83). No significant change in CDR was observed, with a plasma cortisol Pm/am ratio of 0.99 ± 0.14 at baseline compared to 0.94 ± 0.07 at follow-up. Three out of 4 partial responders (patients 5, 14, and 16) showed improvement in weight and waist circumference at follow-up. Blood pressure control improved in 2 patients (no. 14 and 16). In 1 patient (no. 5), blood pressure was normal at baseline and remained unchanged throughout the study period. One partial responder (patient 10) showed worsening of all these clinical parameters (Supplementary Table S2) [36].

### Nonresponders

In the nonresponder group, UFC increased in all 4 patients (no. 2, 4, 12, and 15) immediately after ketoconazole discontinuation despite increased doses of octreotide up to 30 mg/month (Fig. 2C). In 3 (patients 2, 4, and 15) out of 4 nonresponders, UFC levels were unchanged during follow-up compared to baseline. In 1 patient (no. 12), the UFC level at follow-up was doubled compared to baseline. The cortisol Pm/am ratio did not improve during treatment (*P* = .20). Three (patients 2, 4, and 12) of 4 nonresponders lost weight at follow-up. Blood pressure remained unchanged in all 4 patients (Supplementary Table S2) [36].

### Ketoconazole-Cabergoline Combination Treatment

Finally, in 2 patients with baseline UFC levels of 2.31 and 1.55 ULN, hypercortisolism could not be controlled with ketoconazole monotherapy. The addition of cabergoline did not result in a normalization of UFC. Patients remained uncontrolled during the study period, and an alternative treatment modality was implemented.

### In Vitro Studies

Corticotroph tumor tissue was available for the assessment of *SST2* mRNA in 4 patients: 2 responders (patients 8 and 13), 1 partial responder (patient 5), and 1 nonresponder (patient 15) (Fig. 3) who underwent transsphenoidal surgery after the trial. Of these, all but 1 patient had normalized UFC levels before surgery. The nonresponder (patient 15) had slightly elevated UFC (1.22 times the ULN). *SST2* mRNA expression was highest in the tissue of the 2 responder patients (patient 8, relative expression 0.803 and patient 13, 0.216 normalized to *hprt*). It is important to highlight that these *SST2* mRNA expression values (0.803 and 0.216) were comparable to *SST2* expression in GH-secreting tumors (mean of 0.27 ± 0.30, normalized to *hprt*, n = 10) as we have previously published [32]. Corticotroph tumor tissue of the partial responder (patient 5) also expressed *SST2*, albeit at a lower level than the 2 responder patients (0.146 normalized to *hprt*). *SST2* expression was low in corticotroph tumor tissue of the nonresponder (0.08 normalized to *hprt*).

**Figure 4.**
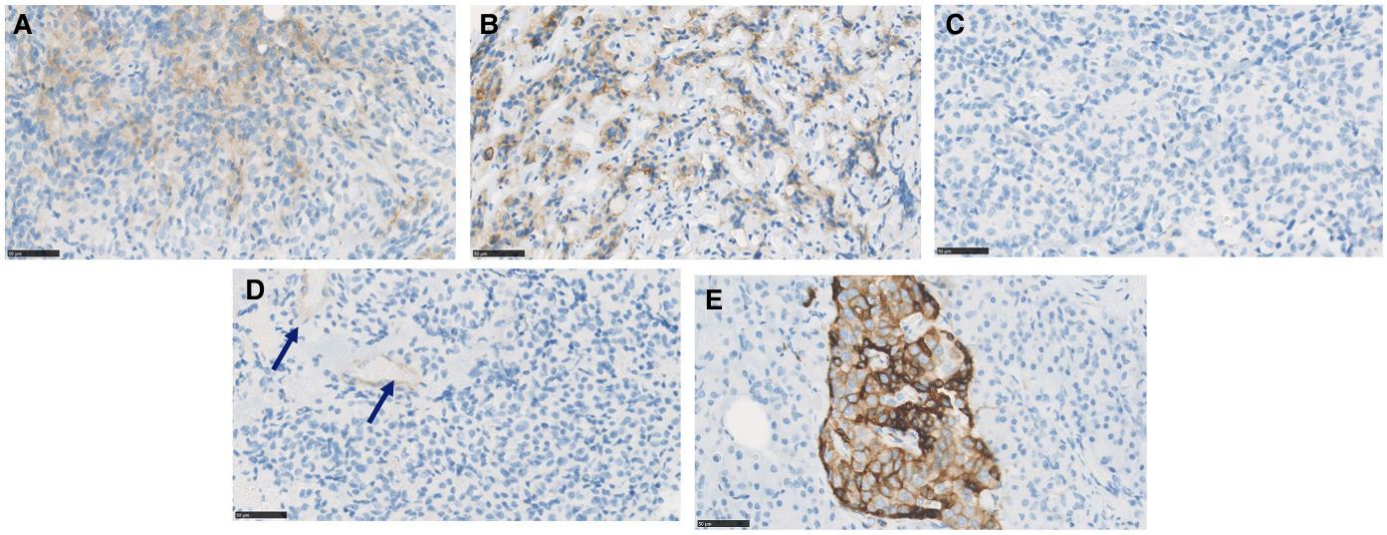
Representative immunohistochemistry of SST2 in corticotroph tumors. Representative photomicrographs of SST2 immunohistochemical staining in formalin-fixed paraffin-embedded tissue sections of 4 corticotroph adenomas of patients included in this study. (A) Adenoma patient 7 (responder) (IRS 4); (B) adenoma patient 5 (partial responder) (IRS 8); (C) adenoma patient 10 (partial responder) (IRS 0); (D) adenoma patient 15 (nonresponder) (IRS 0). (E) Positive control SST2 staining in human pancreatic islets. In most corticotroph adenomas, small blood vessels were SST2 positive (see arrows in panel D). Abbreviation: IRS, immunoreactivity scoring system.

Paraffin-embedded tissue was available for IHC in 4 patients, of which 1 was a responder (patient 7), 2 were partial responders (patients 5 and 10), and 1 was a nonresponder (patient 15). Both mRNA and protein expression were available and assessed for 2 patients who were a partial responder (patient 5) and a nonresponder (patient 15). Before surgery, UFC levels were slightly elevated in 1 partial responder (patient 10) and the nonresponder (patient 15; UFC 1.17 and 1.22 times the ULN, respectively) but normal in the other patients. The IRS for SST2 was higher in the responder compared to the nonresponder patient (IRS 4 and 0, respectively) (Fig. 4). One partial responder (patient 5) had a high IRS for SST2 (IRS 8) with more than 80% of the adenoma cells staining positive for SST2. The second partial responder (patient 10) had no adenoma cells staining positive for SST2 (IRS 0). This patient had slightly elevated UFC levels prior to surgery (described earlier).

## Discussion

Selective downregulation of SST2 expression in corticotroph tumor cells by high cortisol levels is thought to impair the efficacy of SST2 preferring somatostatin analogs in the treatment of CD [29, 30]. The transcriptional regulation of SST2 is modulated by glucocorticoids (GC), as it was demonstrated that GC inhibits SST2 promoter activity through GC-responsive elements, resulting in a decrease in SST2 expression [29]. Because this process may be reversible, we examined in a prospective pilot study whether lowering cortisol production with ketoconazole can enhance inhibition of ACTH secretion via subsequent treatment with octreotide in patients with CD. The existing literature of clinical studies using octreotide in CD consisted of case reports (Table 1). This is the first prospective study to evaluate the clinical efficacy of octreotide in CD. Our data may indicate that the sequential strategy treatment with ketoconazole and octreotide can induce sustained biochemical remission in a subset of patients with mild CD.

Several in vivo and in vitro studies provide evidence that SST2 expression in corticotroph tumor cells can recover after suppression of cortisol production or antagonizing cortisol action [27, 33, 38, 39]. As mentioned, we previously demonstrated that SST2 expression is higher in corticotroph tumors of patients operated under controlled cortisol production compared to those of patients with hypercortisolism at the time of operation [32]. However, SST2 expression was only significantly higher at the mRNA level but not at the protein level. Evidence that SST2 expression can also increase at the protein level was provided by case descriptions of 2 patients with ectopic ACTH syndrome [38]. In both patients, the source of ectopic ACTH production was initially occult with negative somatostatin receptor scintigraphy. However, after treatment with mifepristone, antagonizing the effects of cortisol at a tissue level, somatostatin receptor scintigraphy could identify a neuroendocrine lung tumor in both patients, indicating SST2 protein expression. This was recently confirmed by similar observations in 2 patients with an ACTH-producing neuroendocrine lung tumor [39]. In addition, in vitro studies with the selective GC receptor antagonist relacorilant demonstrated the reversal of GC-induced downregulation of SST2 expression in the AtT20 corticotroph tumor cell line [39]. Finally, indirect evidence comes from an older preliminary study in which a further decrease in UFC levels was observed in 4 ketoconazole-treated patients after the addition of octreotide. The ketoconazole dose could subsequently be reduced in 3 patients [27].

The sequential treatment with ketoconazole and octreotide in the present study led to a partial or complete response in 7 out of 11 patients, with 3 of them exhibiting sustained biochemical remission throughout the follow-up period. At the first stage, ketoconazole monotherapy led to normal UFC levels in 79% of the cases. This efficacy is higher compared to previous studies that reported an efficacy of approximately 50% to 60% and can be explained by the fact that the majority of patients had mild hypercortisolism [11, 40-43]. Additionally, the clinical benefit of controlling cortisol secretion was evident with the observed weight loss in most responders to ketoconazole.

Subsequently, the combined therapy (ketoconazole and octreotide) was able to maintain biochemical remission according to UFC levels. No additive effect was observed with add-on treatment during a period of 2 months of combined ketoconazole-octreotide therapy. Following this stage, ketoconazole was stopped, and treatment was continued with octreotide monotherapy that was able to maintain normal UFC levels in 3 (27%) patients. Since the majority of reported cases using octreotide for CD treatment showed failure to induce biochemical remission, as summarized in Table 1, these results suggest that, in a subset of patients, ketoconazole-induced biochemical remission may have indeed led to upregulation of SST2 with subsequent effectiveness of octreotide.

This is supported by the observed dose dependency in the response to octreotide in both the ketoconazole-octreotide combination phase and the octreotide monotherapy phase. In 2 patients treated with ketoconazole and octreotide, UFC levels increased above the ULN after initial normalization but returned to normal values after a dose increase of octreotide. In 2 of the 3 responders to octreotide monotherapy, an increased dose of octreotide was required, and effective, after an initial increase in UFC levels was observed following ketoconazole discontinuation. Of note, given the size of the present study, a starting dose of octreotide cannot be defined based on our data. A previous study showed that ketoconazole can inhibit ACTH secretion in rat corticotroph cells in vitro; therefore, central effects of ketoconazole in vivo cannot be fully excluded [44]. However, sustained normal UFC levels under octreotide monotherapy in 1 responder patient and the dose-dependent response to octreotide in 2 other responders suggest that a central residual effect of ketoconazole is unlikely to explain the response to octreotide.

Interestingly, among the 3 patients considered as responders based on the UFC levels, clinical improvement was observed in 2 patients in terms of weight loss, waist circumference, and blood pressure control. Notably, the small sample size and limited follow-up reduce our ability to assess the long-term clinical impact of the ketoconazole-octreotide sequential strategy.

A common feature of the 3 patients in whom the strategy was most effective is that they had mildly elevated UFC levels at baseline as compared to patients in whom the strategy failed. This is similar to what was observed in studies with another somatostatin analog, pasireotide, which has been shown to be more effective in patients with less severe hypercortisolism [18, 19]. It is important to acknowledge that octreotide has a safer side-effect profile as compared to pasireotide, which is known to induce or worsen hyperglycemia via inhibition of incretin release. Octreotide could, therefore, be a potentially interesting option to maintain remission in (mild) CD after induction therapy with a steroid synthesis inhibitor [31].

When analyzing the 4 nonresponders and 4 partial responders in the trial, in whom, despite ketoconazole effectively reducing cortisol secretion, octreotide monotherapy was unable to maintain normocortisolism, the reasons for a failed response remain speculative. It is possible that because of more severe baseline hypercortisolism in these patients, as compared to the responders, a longer duration of biochemical remission is necessary in order to restore SST2 expression to adequate levels. Alternatively, corticotroph tumors in these cases may not express an adequate amount of SST2, regardless of the cortisolemic state.

Expression of SST2, defined by either immunohistochemical or mRNA level, is positively correlated with octreotide efficacy in GH-secreting tumors [45, 46]. Accordingly, the 2 responder patients to octreotide in whom cortisol levels were normalized before surgery had higher SST2 mRNA expression compared to partial/nonresponder patients, and these SST2 mRNA expression levels were comparable to the levels in somatotroph tumors [32]. The strategy of lowering cortisol levels to increase SST2 expression may have contributed to octreotide efficacy in these patients. Accordingly, an intermediate level of SST2 mRNA was found in the partial responder, whereas the nonresponder patient had a low level of SST2 mRNA. Regarding SST2 protein expression, a responder patient had an intermediate level of SST2, which may explain the efficacy of octreotide treatment. Consistently, the nonresponder patient to octreotide had no SST2 expression as determined by IHC, which may be explained by preoperative hypercortisolism with concomitant effects on SST2 expression level (mRNA and protein). The partial responders had contradictory results, 1 with high and the other with no SST2 expression by IHC. The partial responder with no SST2 protein expression also had high cortisol levels, which may have contributed to this negative result.

The present study needs to be analyzed in light of its inherent limitations. The single-arm design and small sample size, ie, 14 patients with 3 full responders to octreotide, only permits a descriptive analysis without more robust statistics. This is an important limitation, even considering that, given the rarity of CD, the existing literature consists mostly of case reports. Additionally, the period of 9 months of follow-up limited our ability to more thoroughly appreciate the potential clinical benefits associated with the reduction of UFC levels observed with the sequential treatment strategy tested in this trial. The protocol included ACTH measurements every 3 months, so the impact of octreotide treatment on ACTH secretion was not evaluated in the present study. Finally, in corticotroph tumors, only in selected cases sufficient appropriate tissue was available for mRNA and protein analysis. Generally, adenoma tissue pieces in CD are (very) small, representing a challenge to obtaining enough tissue for molecular studies. This is a well-known problem with respect to in vitro studies with corticotroph adenomas.

In conclusion, a treatment strategy consisting of sequential treatment with ketoconazole to lower cortisol levels, followed by octreotide to maintain biochemical remission, may be effective in a subset of patients with mild CD. Additional studies with longer follow-up are warranted to confirm the long-term efficacy of this strategy for the medical treatment of CD.

## Data Availability

Some or all datasets generated during and/or analyzed during the current study are not publicly available but are available from the corresponding author on reasonable request.

## Clinical Trial Information

Dutch Trial Register nr. NL37105.078.11.
